# Silicon Metalens Fabrication from Electron Beam to UV-Nanoimprint Lithography

**DOI:** 10.3390/nano11092329

**Published:** 2021-09-07

**Authors:** Angela Mihaela Baracu, Marius Andrei Avram, Carmen Breazu, Mihaela-Cristina Bunea, Marcela Socol, Anca Stanculescu, Elena Matei, Paul Conrad Vaagen Thrane, Christopher Andrew Dirdal, Adrian Dinescu, Oana Rasoga

**Affiliations:** 1 National Institute for Research and Development in Microtechnologies-IMT Bucharest, 126A, Erou Iancu Nicolae Street, 077190 Voluntari, Romania; angela.baracu@imt.ro (A.M.B.); andrei.avram@imt.ro (M.A.A.); 2 National Institute of Materials Physics, 405 A, Atomistilor Street, P.O. Box M.G. 7, 077125 Magurele, Romania; carmen.breazu@infim.ro (C.B.); mihaela.bunea@infim.ro (M.-C.B.); marcela.socol@infim.ro (M.S.); sanca@infim.ro (A.S.); elena.matei@infim.ro (E.M.); 3 SINTEF Microsystems and Nanotechnology, Gaustadalleen 23C, 0737 Oslo, Norway; paul.thrane@sintef.no (P.C.V.T.); christopher.dirdal@sintef.no (C.A.D.)

**Keywords:** EBL patterning, cryogenic etching process, stamp fabrication, UV-NIL patterning

## Abstract

This study presents the design and manufacture of metasurface lenses optimized for focusing light with 1.55 µm wavelength. The lenses are fabricated on silicon substrates using electron beam lithography, ultraviolet-nanoimprint lithography and cryogenic deep reactive-ion etching techniques. The designed metasurface makes use of the geometrical phase principle and consists of rectangular pillars with target dimensions of height h = 1200 nm, width w = 230 nm, length l = 354 nm and periodicity *p* = 835 nm. The simulated efficiency of the lens is 60%, while the master lenses obtained by using electron beam lithography are found to have an efficiency of 45%. The lenses subsequently fabricated via nanoimprint are characterized by an efficiency of 6%; the low efficiency is mainly attributed to the rounding of the rectangular nanostructures during the pattern transfer processes from the resist to silicon due to the presence of a thicker residual layer.

## 1. Introduction

Metasurfaces enable the possibility of transition from bulky and heavy optical components toward small, lightweight and cheap ones [1,2,3,4,5,6]. This transition, in combination with the fast evolution of sensor technologies based on light-matter interactions, is fueling the interest in producing ultrathin (around 1 µm) optical lenses called metasurface lenses (metalenses) [7]. Due to their design, metalenses offer a unique control over the electromagnetic field. Thus, metalenses consist of thin planar surfaces upon which a single layer of periodic, quasi or aperiodic arrays of (nano)patterns (also called nanostructures or nanoantenna) with different geometries and subwavelength spatial resolution are fabricated in order to achieve the desired spatial profile of optical phase or spectral response [8,9,10,11]. Therefore, as part of their compact dimensions, an advantage over the classical lenses is connected with their ability to shape light either actively or passively by the manipulation of the amplitude, phase or polarization, outperforming them in several areas [12,13,14,15,16]. Generally, the fabrication of the metalenses is based on standard planar microfabrication techniques focused on a specific domain, from ultraviolet (UV) to near-infrared (NIR) due to the nature of the used materials in order to obtain diffraction-limited performances and higher efficiencies [4,17,18,19,20,21].

For the micro-/nano-pattern fabrication, the photolithography [9,22,23] and the electron beam lithography (EBL) [17,24] are the most used techniques. EBL provides good patterning results for nanoscale features (called “direct writing technique”). EBL consists of patterning the desired shape directly on the negative or positive resist with a Gaussian electron beam with a diameter in the angstrom scale. However, this technique cannot be applied to low-cost and large-area manufacturing because the transfer of the full pattern onto the resist requires longer processing times (the beam is focused on a single point of pattern at a time), implying high operating costs [5,17,25,26,27,28,29].

As an alternative to both EBL (expensive and time consuming) and photolithography (limited by the light diffraction), the nanoimprint lithography (NIL) technique assures large-scale high pattern resolution in seconds. Usually, the NIL term has been used for various types of techniques, starting with the hot-embossing (thermal) and roll imprint process up to the reverse imprint or ultrasonic nanoimprint lithography [30,31,32,33,34], but in principle, it is connected with the thermomechanical or UV curing processes [35,36,37].

In the last decade, the UV-NIL technique has been developed in order to provide a cheap and large-scale fabrication alternative for industry [4,5,11,20,38,39,40,41]. The NIL-technology implies two fundamental aspects: (i) the basic research and (ii) the applied research. The basic research is connected with all the fabrication steps applied to realize micro-/nano-patterns: process, tool, master, stamp material and resist. One of the drawbacks compared to the EBL technique is the need of a mask. Usually, in the mask fabrication, the EBL process is followed by the etching procedure. In order to decrease the costs and time in the UV-NIL technology, the initial mask, called “master template” (or simply named master) is used to create negative replicas (called “stamps” or “molds”) presenting the inverted EBL patterns. These stamps are further utilized in the imprint process such as for masks, and therefore, the life-time of the master is extended. Thus, a key feature of the process flow is connected with the realization of the master template [42]. Although there are several studies based on the nanoimprint process, the issues regarding the defect mechanism have not been completely solved [43,44]. The common defect mechanism that appears in the NIL processes is connected with the demolding step, in which the stamp has to be detached from the cured resist. Generally, various antisticking solutions based on silane compounds are used in order to increase the contact angle to obtain a hydrophobic stamp surface. Still, the defects generated by the polymer sticking on the stamp surface are present due to the interfacial forces (adhesion and friction forces) between the resist and the stamp material. The interfacial forces are strongly linked to the quality of the stamp (design, roughness, antisticking layer and material), meaning by the quality of the master, resist material but also by the mechanical characteristics of the used UV-NIL tool [43]. In addition, these forces are dependent on the residual stress that appears during the UV irradiation due to the shrinkage of the resist that makes the stamp to adhere more to the resist surface [44].

For this reason, more studies are required for extending the metasurface lens research domain and their commercial applications using the UV-NIL technique. Thus, for the optimization of the master and the stamp, the fabrication processes are still challenging in order to (a) preserve the fidelity of the (nano)patterns and (b) facilitate the stamp release by tuning the etching parameters. In this context, the purpose of the present study is (i) to create a master by different approaches of the etching process and (ii) to provide a stamp replica in poly- (dimethyl siloxane) (PDMS) of a metasurface lens design operating in the near-infrared domain, which can be further utilized for the NIL process. Thus, both techniques, EBL (for master fabrication) and UV-NIL, a pattern transfer method using mechanical deformation by the light pressing of the “UV-transparent nanopatterned stamp” against the substrate coated with a liquid resist that solidifies due to the ultraviolet radiation are used in this work [5,45,46].

Compared to the works already reported [1,4,5,9,14,47], this study presents all the fabrication steps necessary to realize diffraction-limited dielectric metalenses for NIR domain, starting from the EBL and finishing with the classical UV-NIL technique instead of the stepper technique using flexible backplane for the soft stamp material, pointing out the issues that we encountered. The main goal is to achieve a base technological chain that can be further utilized in the fabrication of metalenses by UV-nanoimprint technique without the need for buying “master templates” that are usually expensive.

For the master manufacturing, an EBL system was implied in writing the patterns on a positive electron resist of 200 nm thickness. Since one of the requirements of the process fabrication is to obtain high aspect ratio (HAR) structures, special attention was given to the transfer of the patterns on the silicon wafer. Therefore, different masking layers and recipes were used in order to optimize the process for reliable pattern transfer. The quality of the master and metalenses made by the abovementioned process was optically analyzed. Their efficiencies reached up to 45% compared to 60% expected by simulation.

We obtain a master and fabricate the transparent stamps for the further UV-NIL process using a common UV-lamp for the PDMS curing and two experimental approaches. In order to avoid the demolding or diffusion problems, compatible stamp material, antisticking layer and resist were used in the fabrication recipe, with all these compounds being easily used in a research laboratory. The obtained diffraction limited spot profile is similar with the one of the metalenses fabricated by EBL. The low efficiency of the metalenses linked to the edge rounding of the rectangular nanopatterns that in turn is responsible for a decrease in the cross-polarization can be improved by further optimization of the processes.

## 2. Materials and Methods

### 2.1. Lens Design Methodology

**Figure 1:**
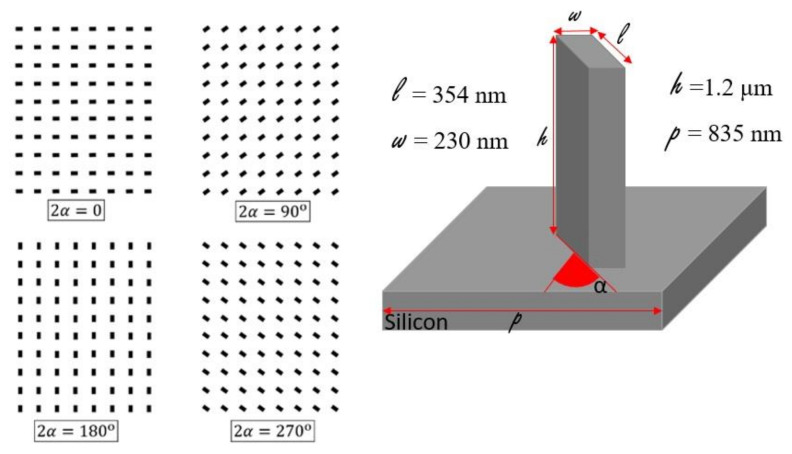
Rectangular pillars acting as phase shifters on circular polarized light through the geometrical phase principle. The relative rotation angle α imposes a relative phase difference equivalent to 2α to the cross-polarized circular polarization state.

The metastructure is designed to impose pointwise phase shifts *ϕ(r)* to the transmitted field at each radial distance r from the center of the metasurface, which corresponds to the phase function of a lens:

(1) $$\phi \left(r\right)=-\frac{2\pi }{\lambda }\left(\sqrt{r^{2}+f^{2}}-f\right),$$

where *λ* is the wavelength of the field and f is the distance from the metasurface substrate to the point where the field should be focused to a diffraction-limited spot along the surface normal [48,49]. We have chosen the geometric phase principle [14,21,50,51] for application of the desired phase shift. To this end, we propose a metasurface structure consisting of rectangular pillars designed to act as a half-wave plate (details further below), leading to the conversion between left and right circular polarization states of the incident field. Supposing that the rectangular pillars are placed as an identical array with a rotation angle α relative to the lattice vectors (see Figure 1) and that the incoming field is right circularly polarized $\left|E_{in}\rangle \right.=\;\left|R\right.\rangle$, then it can be shown that the transmitted field can be expressed as

(2) $$\left|E_{out}\right.\rangle =t_{x}e^{-i2\alpha }|L\rangle ,$$

where $t_{x}$ is the complex transmission coefficient for linear polarized light along the *x*-axis and that the metasurface design ensures $t_{y}=-t_{x}$ (the corresponding coefficient for linear polarized light along the orthogonal axis is equal, apart from a sign change, thereby realizing a half-wave plate) [4]. It can be seen that the rotation angle α of the rectangular pillars imposes a phase 2α to the output field. From the expression above, it may be noted that the efficiency of this half-waveplate metalens is given by the transmissivity ${\left|t_{x}\right|}^{2}$. If scattering and reflections caused by the metasurface structure are disregarded, a simple transmissivity estimate is that of the plane substrate.

**Figure 2:**
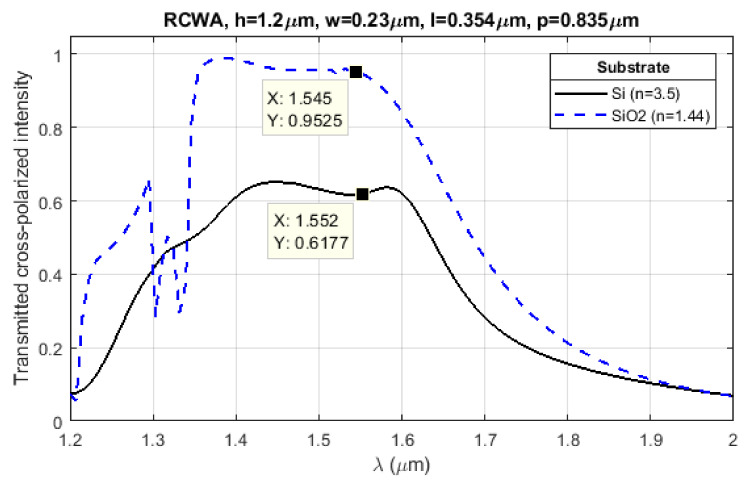
Rigorously coupled wave analysis (RCWA) simulations of rectangular silicon pillars on two different substrate materials: silicon and silicon dioxide (e.g., a quartz or fused silica wafer). The difference in efficiency is largely explained by differences in transmissivity from the substrate to air due to their differences in refractive index: 3.5 for Si and 1.44 for SiO_2_ for NIR.

In our case, a silicon substrate is used for which transmission from silicon to air is on the order of 69% for λ = 1.55 µm. Similarly, if instead a quartz substrate is used, the corresponding transmissivity can be increased to 97% (efficiencies for metalenses utilizing quartz substrates are often on the order of 80% or larger [9,21,52]). The increase in theoretical efficiency upon switching from a silicon substrate to a silicon dioxide (SiO_2_) substrate is shown in Figure 2. Apart from process imperfections, the discrepancy from the simple efficiency estimate offered here can be attributed to scattering and reflections caused by the metasurface structure. In the case of silicon substrates considered here, simulations indicate an upper theoretical efficiency of around 60%.

The above discussion motivates the commonly used design procedure for the metasurface lenses (metalenses) [51]. To achieve pointwise phase control according to $\phi \left(r\right)$ over the metasurface, we chose to rotate the pillar within each unit cell according to $\alpha \left(r\right)=-\phi \left(r\right)/2\;.$ Note that the above discussion was based on identical arrays of structures, whereas we now allow for each rectangular pillar to be rotated differently from its neighbors, making the proper description of the transmitted field more complicated (taking diffractive scattering and variation of neighbor interactions into account). Nevertheless, the description is sufficiently accurate for our purposes [4].

The dimensions of the rectangular pillar metastructure necessary to achieve the desired conversion of handedness of circularly polarized light was found by sweep simulations relying on rigorously coupled wave analysis (RCWA) and the finite difference time domain method (FDTD). The RCWA method was used in the GD-Calc implementation to simulate a unit cell consisting of an individual pillar, and 10 diffraction orders were used. The FDTD method was used in the OptiFDTD implementation for which periodic boundary conditions around the unit cells (orthogonal to the optical axis), and perfectly matched layers at the beginning and the end of the optical axis were used. For the FDTD method, a nonuniform mesh grid was used to allow for a fine mesh near to the Si pillars, which is necessary for simulating process imperfections such as tapering and rounding effect of the pillars. For the directions orthogonal to the pillar heights (i.e., orthogonal to the optical axis), the FDTD mesh grid interspacing varied between 3 nm≤Δx,Δy≤6 nm, while along the optical axis the mesh grid interspacing varied between 3 nm≤Δz≤10 nm. The simulations assume the source was placed within the silicon substrate. For the target wavelength λ = 1.55 μm, a source pulse width of FWHM of $6.84\cdot 10^{-15}s.$ and a simulation time of 25,000 timesteps of $\Delta t=5.55\cdot 10^{-18}s.$ each were used. To cover the whole wavelength, bandwidth of interest multiple simulations were conducted for different center wavelengths (for which the FWHM bandwidth varied slightly).

The details of these simulations are given in [4]. It was found that the target dimensions of h = 1200 nm, width w = 230 nm, length l = 354 nm and periodicity *p* = 835 nm give full cross-polarization at the target wavelength of λ = 1.55 µm.

### 2.2. Experimental Approach

**Figure 3:**
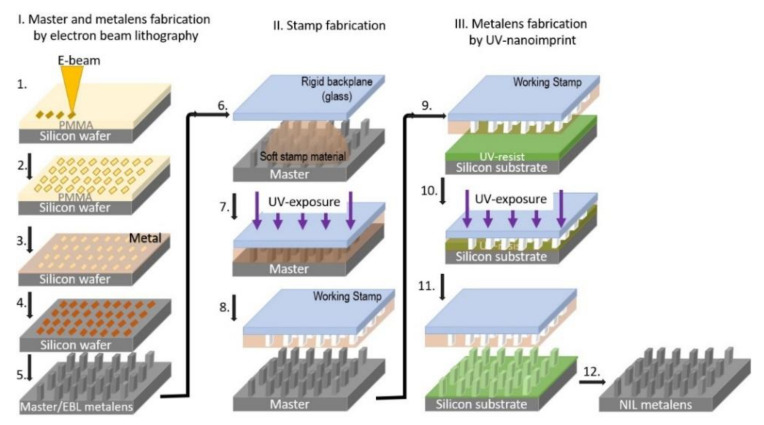
Sketch of the experimental steps used to pass from the EBL to UV- NIL: 1. Resist deposition and further electron beam exposure; 2. Developing of the exposed PMMA resist; 3. Masking layer deposition; 4. Lift-off; 5. Silicon cryogenic etching process with O_2_ and SF_6_ and reactive ion etching to completely remove the masking layer; 6. Dropcast of the soft stamp material (UV-PDMS) on the master template; 7. UV-curing of the soft stamp material; 8. Demolding the rigid backplane with the soft stamp material on top (working stamp) from the master template; 9. Alignment of the working stamp and the resist-coated substrate; 10. Contact between the working stamp and resist followed by resist curing; 11. Detachment of the working stamp from the patterned substrate; 12. Residual layer removal by reactive ion etching, silicon cryogenic etching process with O_2_ and SF_6_ and reactive ion etching to completely remove the resist layer.

In order to have flexibility in the IR metalens process fabrication according to the further needed designs, we tried to put the base of a chain of processes (presented in Figure 3) that could help us reduce the time and costs.

#### 2.2.1. UV-NIL Master and “EBL Metalens” Fabrication

The UV-NIL master fabrication and “EBL metalenses” were carried out on 4 inches (100) *p* type silicon wafers, with a resistivity of 5–10 Ω·cm and 525 µm thickness, purchased from Siegert Wafer. The periodic arrays of nanostructured metasurface were performed using e-beam lithography and cryogenic-deep reactive-ion etching (DRIE) processes. The bottom-up approach to build up the nanoscale structures involves the evaporation of metal film followed by the lift-off process.

Two different metals and a SiO_2_ thin films were proposed as masking layers for the silicon dry etching process. In terms of fabrication steps, the technology implying the SiO_2_-like masking layer is more complex than the one for the metals, due to the fact that it requires 3 supplementary processes: metal deposition and both reactive ion and chemical etching.

The thermal SiO_2_ was chosen as masking layer because it is one of the most employed etch masks used in cryogenic etch processes. Thus, a 100 nm SiO_2_ thin film was grown on the surface of the Si wafer using a thermal oxidation furnace before the e-resist deposition. A layer of positive electron resist poly- (methyl methacrylate) (PMMA)) with a thickness of 200 nm was spin-coated on the SiO_2_-coated wafer and then patterned by a dedicated e-beam equipment (RAITH e_Line). The following parameters were used in the exposure: acceleration voltage—30 kV; beam current—200 pA and clearing dose—300 µC/cm^2^. The proximity effect was corrected by using PECS software module. To reveal the patterns, the irradiated wafers were developed in a mixture methyl isobutyl ketone (MIBK): isopropyl alcohol (IPA) (1:3) for 60 s at 22 °C. A Cr/Au thin film of 30/50 nm was subsequently deposited via e-beam evaporation method, followed by the lift-off process. The etch pattern was transferred from the metallic layer to the SiO_2_ by reactive ion process (RIE) based on trifluoromethane (CHF_3_) and argon (Ar) chemistry using the Etchlab SI 220 (Sentech Instruments, Berlin, Germany) equipment. The Cr/Au thin film was removed by chemical etching after transferring the nanopatterns to SiO_2_.

For the metallic masking layers, the PMMA was deposited directly onto the silicon substrate. The EBL process and development were similar with the ones explained above for the SiO_2_ masking layer. The metallic masking layers were deposited onto the developed PMMA resist. The first metallic mask tested was a thin 50 nm Al layer. This metal was chosen because the potential contamination of the reactor chamber is low due to sputtering by products. The second metallic masking layer was a 30 nm Ti thin layer.

The subsequent silicon etching process was the same for all the masking layers and was performed in an inductively coupled plasma reactive ion etching (ICP-RIE) system, Oxford Instruments Plasmalab 100 Ltd., Yatton, UK, fitted with a liquid nitrogen Dewar, allowing cryogenic etching process at temperatures as low as −120 °C. The final plasma parameters used for the cryogenic process were presented below in Table 1, and the etch rate of the process was approximately 28 nm/s [47].

#### 2.2.2. UV-NIL Stamp Fabrication—Master Replication Using UV-PDMS (KER-4690)

The “master” fabricated by EBL and cryoetching process was further used for fabrication of the stamp for UV-nanoimprint process, employing the UV-silicone rubber -KER-4690 A/B from OEM: Shin-Etsu, supplied by micro resist technology GmbH, Germany. This silicone rubber is a PDMS specially designed to form a nonadhesive layer and to provide a high-definition transfer [53].

The stamps were fabricated on rigid backplanes (4” glass wafers) that were previously cleaned (by ultrasonic cleaning in acetone and IPA for 15 min each and dried with nitrogen). Further, 10 min plasma treatment and 30 min thermal treatment at 200 °C were applied to enhance the surface hydrophilicity.

Simultaneously, the master template was cured for 10 min in oxygen plasma, annealed 15 min at 200 °C, and spin-coated with the filtrated BGL-GZ-83 solution using the following parameters (provided by the seller –PROFACTOR GmbH (Austria)): step I: 1000 rpm for 30 s, Step II: 2000 rpm for 30 s with 1600 rpm/s acceleration ramp. The BGL-GZ-83 is an antisticking layer (ASL) which provides a different, simple solution to decrease the surface free energy of the master or stamp without using the physical vapor deposition of silane compounds.

The glass wafer is kept as it is, without any adhesion promoter. The two components of the PDMS are vigorously mixed together in 1:1 ratio with a plastic spatula for about 10 min. The degassing phase was realized by leaving the mixture almost 2 h to rest. Further, a 1.4 g UV-PDMS solution was poured on the middle of the master over which the coated glass substrate is carefully placed, starting from one edge in such a way that the air is not trapped inside the resin. In order to spread the prepolymer mixture onto the entire master/glass substrate interface due to the capillary forces, the stack is left for 15 min before curing. Further, the stack is exposed to the UV light of a mercury vapor grid lamp (GLF-42) without filters. The curing has carried out in two ways: (i) in a successive irradiation time at 40 mW/cm^2^: 5, 7, 7 and 7 min—until the PDMS was completely cured or (ii) 7 min’ irradiation time at 40 mW/cm^2^, then being left overnight (almost 19 h) at room temperature.

#### 2.2.3. UV-NIL Metalenses Fabrication

The metalenses fabrication by UV-NIL can be divided in 2 major steps as follows: (1) the UV-nanoimprint process which imprints the negative copy of the stamp in a liquid resist sensitive to the UV-light and (2) cryogenic-DRIE process which gives the final form of the metasurface, etching the silicon wafer.

For the UV-NIL process, the same type of silicon wafers was used, similar to the case of the master. The substrates were cleaned in acetone and IPA for about 10 min each, and then a short oxygen plasma treatment for 10 min was applied in order to obtain the hydrophilic surface. The mr-NIL210 resist [54] at two standardized versions—200 and 500 nm—was used after prior deposition of an adhesion layer mr-APS1 (acquired both from micro resist technology GmbH, Germany).

The spin-coating process parameters were the ones provided by the supplier. The two standardized versions should give at 3000 rpm for 60 seconds, a film thickness of 200 nm, respectively, 500 nm. The initial tests were made using the 200 nm resist but working with a stamp with h = 1.2 µm. After the first preliminary results, we decided to work with a thicker resist in order to completely fill the stamp cavities.

The nanoimprint was carried out with an EVG 620 mask aligner working at a constant time exposure mode and hard and vacuum contact for better results as indicated by the equipment supplier. The exposure was taken at 22 mW/cm^2^ for 100 s, which means a UV irradiation of 2200 mJ, with a vacuum contact of 50 mbar for the samples made with 200 nm mr-NIL210 and of 150 mbar for the 500 nm mr-NIL210.

Prior the cryogenic etching of the metalenses, the residual layer (RLT—the resist layer thickness that remains between the resist rectangles) has to be removed. The RLT removal was performed using a reactive ion process (RIE) in the Etchlab SI 220 (Sentech Instruments, Berlin, Germany) with the following parameters: 150 mTorr pressure, 200 W ICP power and different values for the O_2_ flow. It was found that the optimal time for the residual layer removal was 50 seconds. The pattern transfer from the mr-NIL210 resist to silicon was achieved by using the same cryogenic parameters mentioned above (see Table 1).

#### 2.2.4. Optical Measurements of the Metalenses

The characterization setup is described in detail in [4] and consists of a collimated and stabilized laser beam with a wavelength 1550 nm, which was passed through a left-handed circular polarizer and a circular aperture with 0.9 mm diameter before being focused by the metalens. The focal spot was imaged using a x20 NIR infinity corrected microscope objective, tube lens and NIR camera. To increase contrast, a right-handed circular polarizer was placed in reverse between the microscope objective and tube lens, thus filtering out left-handed circular light which had not been converted to right-handed light by the metalens.

## 3. Results and Discussion

### 3.1. Master and “EBL Metalens”

For the master/metalens fabrication by EBL, in order to achieve an etch depth of 1.2 µm, a SiO_2_ thin film and two different metals were proposed as the masking layer for the silicon dry etching process. The purpose was to determine the selectivity of the etch process, as well as for the reliable transfer of the designed nanoscale patterns from the mask to the etched structures. In this case, resist masks were excluded due to the shrinkage caused by low substrate temperatures resulting in distorted patterns.

The main advantages of using the cryogenic etching process over the room temperature Bosch etch method are the single step process with smoother sidewalls (no scallops) and the exceptional profile control for nanostructures. The cryogenic etching process is a continuous process of using O_2_ for sidewall passivation and SF_6_ as an etchant. At temperatures below −100 °C, the O_2_ in the plasma reacts with F* radicals and condenses on exposed Si surfaces creating a thin oxifluorosilicate barrier which prevents chemical etching of the substrate. This barrier is removed from horizontal surfaces by ions from the plasma, which are accelerated toward the substrate, allowing F* to react with the Si to form SiF_4_ which is volatile. By balancing the O_2_ gas flow versus plasma loading, it is possible to obtain the profile control of the etched nanostructures. High flows of O_2_ lead to positive profiles (trapezoidal shapes), while low flows result in accentuated undercutting. Because the structures resulted by plasma etching are influenced by many factors, all processes were optimized for this specific equipment and application.

**Figure 4:**
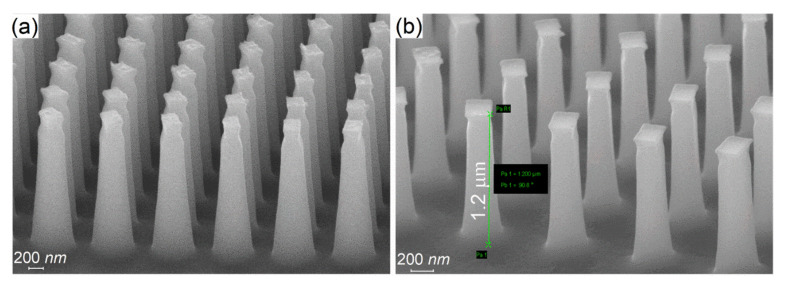
Patterned silicon after cryogenic etching process using (**a**) a deficient and (**b**) an optimal O_2_ flow.

Process optimization was focused on two aspects: the vertical profile of the nanopillars and masking layer. To optimize the vertical profile of the pillars, the O_2_ flow was adjusted in order to obtain near-vertical profiles. The experimental investigations showed that the optimal O_2_ flow was 8 sccm. In Figure 4, we present the cross-section SEM images of pillars etched with deficient O_2_ flow, resulting in a tapered profile, and with optimal O_2_ flow, resulting in a vertical profile.

The use of the thermal silicon dioxide mask ended up to not being suitable for optical applications, since the RIE process used for the oxide etching results in an increased surface roughness, which is further accentuated during the cryogenic process. Therefore, further process optimization was conducted using the two metallic masking layers.

**Figure 5:**
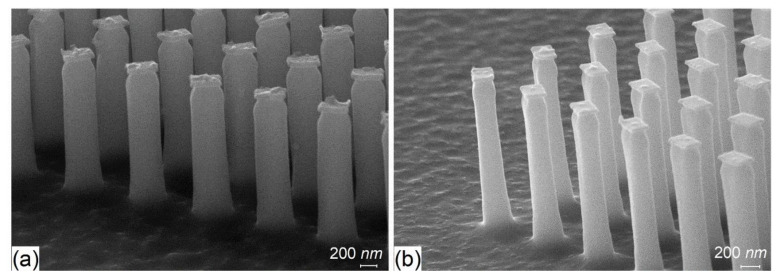
E-beam rectangular nanostructures patterned in the: (**a**) Al and (**b**) Ti films.

After patterning the Al masking layer and etching the Si substrate, we found that structures have an irregular pattern, most likely due to the large grain size of the metal which prevents the accurate transfer of the patterns from using a lift-off process. By replacing the Al masking layer with the 30 nm thin Ti layer, the patterns of the etched structure were significantly improved, resulting in smoother edge lines (see Figure 5).

**Figure 6:**
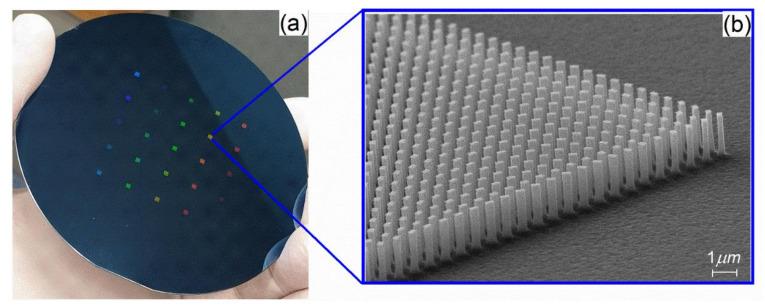
(**a**) Four inches Si master wafer containing nanopatterned metasurfaces via e-beam lithography and cryogenic etching method; (**b**) detail of the metasurface area containing silicon rectangular nanopillars with straight profiles and smooth sidewalls.

The rough surface and tapered profile of the nanostructures are expected when using the conventional top-down approach [21], especially when the pillar height is over 1 µm, but when using metallic layers, the tendency to have pillars with a conical shape decreased significantly (Figure 5a,b).Thus, for the fabrication of the Si “master” and “EBL metalens”, the parameters from Table 1 and the Ti as a masking layer were used. The final optimized master obtained by EBL and cryogenic process etching is presented in Figure 6, where each colored square represents a metalens.

### 3.2. UV-PDMS Stamp

**Figure 7:**
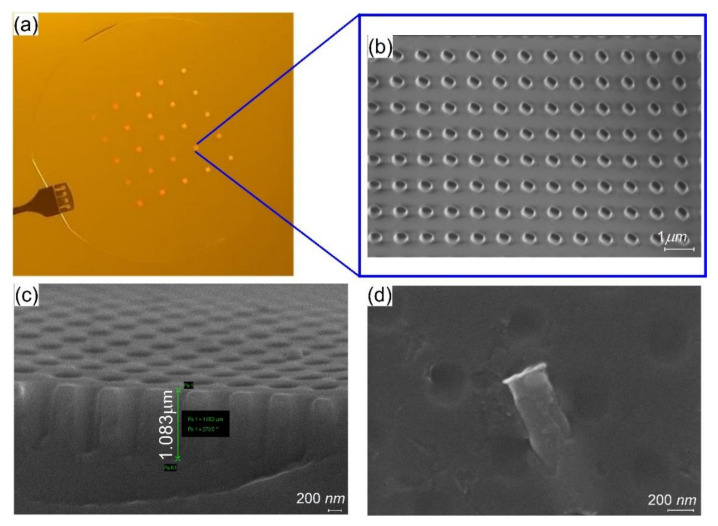
(**a**) Optical image of the fabricated stamp; (**b**) top-view SEM micrograph of the stamp left overnight in contact with the master; (**c**) side-view and (**d**) top-view SEM micrographs of the stamp fabricated in successive irradiation times without being left overnight. It is worth noting that for the (**c**,**d**) images a thin layer of gold has been deposited.

From the SEM analysis made on the fabricated stamps resulted that the one left overnight is the one that presents good pattern fidelity without any visual defects, such as bubbles (see Figure 7a,b).

For the stamp realized by successive UV irradiation, the SEM analysis reveal that the UV-PDMS polymer was partially capable of producing the inverted copy of the metalenses from the fabricated master. The shape of the holes is looking more oval than rectangular, probably due to the fact that the successive curing time before delamination from Si master was too short and thus the cavities have suffered deformation on the direction of the stamp detachment. The height of the holes measured after the thin gold layer deposition was 1.083 µm (1083 nm) with the l = 340.4 nm and w = 224.2 nm. Thus, the deviation from the master is almost of 4.2% in the length and 5.3% in the width. Moreover, in this case, it seems that at the demolding of the stamp from the master step, due to the adhesion forces between them and due to the geometry of the pillars pattern, the fracture occurs that further remains in the stamp silicone rubber affecting the nanoimprint results, although the Si master was treated with ASL (Figure 7d). As a consequence, the use of the master to fabricate other stamps is compromised.

### 3.3. UV-NIL Metalenses

In order to investigate the pattern fidelity of the “UV-NIL metalenses”, SEM analyses were made after: (i) the imprint, (ii) the dry-etching made in order to remove the residual layer and (iii) after the cryogenic-etching process.

**Figure 8:**
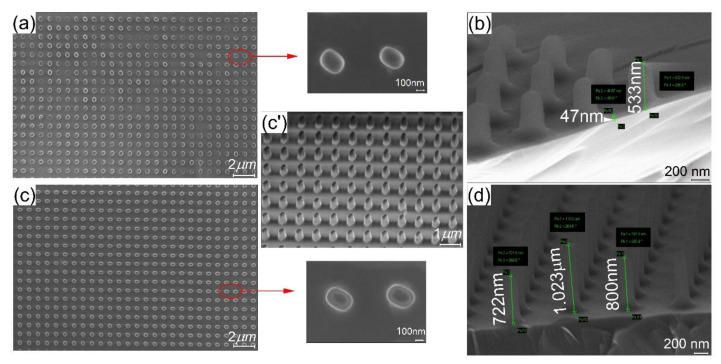
(**a**) Top-view SEM micrograph of the “resist metalens” imprinted at 50 mbar contact pressure and 200 nm resist thickness (**b**) side-view SEM micrograph of the “resist metalens” imprinted at 50 mbar contact pressure and 200 nm resist thickness after residual layer removal using RIE process; (**c**) top-view SEM micrographs of the “ resist metalens” imprinted at 150 mbar contact pressure and 500 nm resist thickness and (**c’**) the respective side view; (**d**) side-view SEM micrograph after residual layer removal of the “ resist metalens” imprinted at 150 mbar contact pressure and 500 nm resist thickness.

The SEM analysis revealed that the NIL process with 50 mbar pressure and 200 nm resist formula was not enough to obtain regular patterns (see Figure 8a). The obtained metalenses were characterized by defects comparatively with the one made at a higher pressure (Figure 8c). If we compare the height of the stamp cavities (Figure 7c) with the height of the resist pillars (~533 nm) (Figure 8b) after incomplete removal of the residual layer, we can conclude that the thickness of the 200 nm resist is not sufficient to completely fill them.

Analyzing the “UV-NIL metalens” fabricated using the 500 nm resist formula from the top and side view (Figure 8c,c’), it can be pointed out that: (i) the height of the patterns is slightly irregular, justified also by the SEM measurements made on the sample after the RIE process (Figure 8d) and (ii) that the rectangular form is not preserved, ending up with a concave shape for the pillars, which is given in this situation by the slightly tapered patterns of the master template. The height fluctuations of the resist pillars between 1023 and 722 nm can be the cause of multiple factors such as: (i) the inhomogeneous stamp surface due to the absence of a perfectly horizontal surface during the contact between the master and the glass carrier, resulting in a difference of material distribution over the wafer, (ii) the thickness gradient of the resist after the spin-coating process, maybe due to the lower humidity (32%), or due to the (iii) inhomogeneous pressure distribution of the equipment.

**Figure 9:**
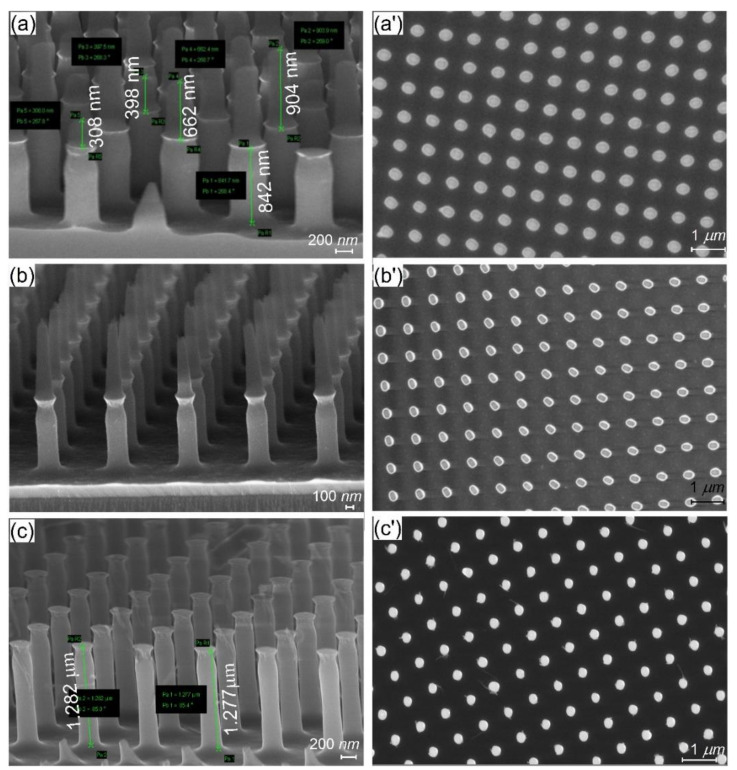
The nanopatterned metalenses via UV-NIL and cryogenic etching method with: mr-NIL 200 nm resist mask side view (**a**) and the respective top view after resist removal using RIE (**a’**); with mr-NIL 500 nm resist mask side view (**b**,**c**) and the respective top view after resist removal using RIE etched (**b’**,**c’**).

A cross-section of silicon nanopillars can be seen in Figure 9a–c. In Figure 9a,b, the pillars without removing the mr-NIL210 masking layer can be seen on top of the silicon patterns, for the 200 nm resist (Figure 9a) and for the 500 nm (Figure 9b) and their top view (Figure 9a’,b’). For the 200 nm resist, the height of the etched pillars was 842 nm, although the mask resist pillars had the heights in the (300,800) nm domain, and unfortunately, the height target dimension of 1.2 µm was not reached in this case due to the incomplete removal of the residual layer (Figure 9a). The shape of the pillars is as we expected from the fabrication step, more oval/rounder than rectangular.

For the metalenses fabricated with 500 nm resist formula, the heights of the etched pillars were 1066 nm (Figure 9b) and around 1280 nm (Figure 9c), surpassing the target dimension with ~80 nm. Unfortunately, by reaching the target high dimension, we have lost the almost rectangular shape that we obtained for the patterns smaller than 200 nm (Figure 9b’), ending up with round thin pillars (Figure 9c’). As a consequence, this rounding leads to a significant decrease in the lens efficiency. The important observation that can be taken from these morphological images is that with the above cryoetching process, the heights of the etched Si pillars are independent of the differences in the heights of the mask resist pillars but can be influenced by the incomplete removal of the residual layer.

### 3.4. Optical Measurements of the Metalenses and Supplementary Discussions

**Figure 10:**
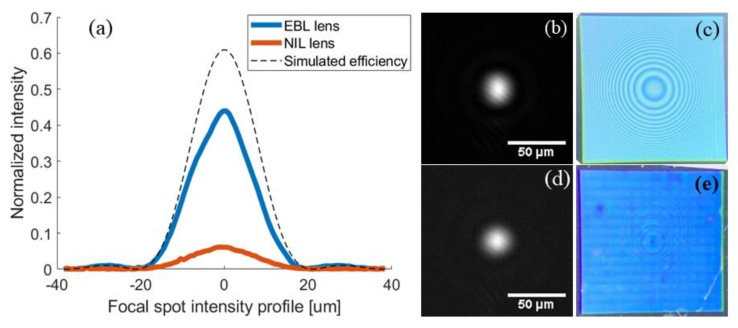
(**a**) Focal spot intensity profile of a metalens made using EBL (blue) and a metalens made using NIL (orange), compared to the theoretical profile for a lens with the designed efficiency (dashed line) for 1550 nm laser light. As discussed in Section 2.1, the less−than−unity efficiency is largely attributed to using a silicon substrate; (**b**) image of the focal spot from the EBL lens; (**c**) optical microscope image of the EBL lens; (**d**) image of the focal spot of the NIL lens, for 1550 nm laser light; (**e**) optical microscope image of the NIL lens with sidelengths 1.5 mm. Note that for the focal spot measurements, a circular 900 µm aperture was placed in front of the lenses.

A total of four metalenses fabricated using EBL as well as using NIL (two for each technique) was optically characterized, and their focal spot profiles were shown in Figure 10. Since, respectively, both EBL metalenses and both UV-NIL metalenses were identical from the focal spot and efficiency point of view, we chose to represent just one for each type.

The focal spot profiles are compared to that of an antireflection coated aspherical lens, and the efficiencies are found to be 45% for the metalenses made directly using EBL, and 6% for the metalenses made using NIL. These efficiency numbers do not include the 31% reflection loss from the backside of the Si substrate. As can be seen in Figure 10, all lenses have the same diffraction limited spot profile, which is a result of the geometric phase design—the phase of the cross-polarized light being decided by the nanostructure orientation, which is accurately reproduced in both the EBL and NIL processes.

Meanwhile, the focusing efficiency is dependent on the length and width of the nanostructures (see Table 2). When achieving the target dimensions, we would expect in the order of 60% efficiency from simulations. However, the analyzed lenses fabricated using NIL, having reached the target high dimensions but with a round top (Figure 9c,c’) and visible surface defects (see Figure 10e), have low cross-polarization efficiency; therefore, the fabrication process has to be optimized for better structure uniformity as well as for achieving final dimensions closer to the target ones—for example, by taking systematic fabrication errors into account in the design process.

**Figure 11:**
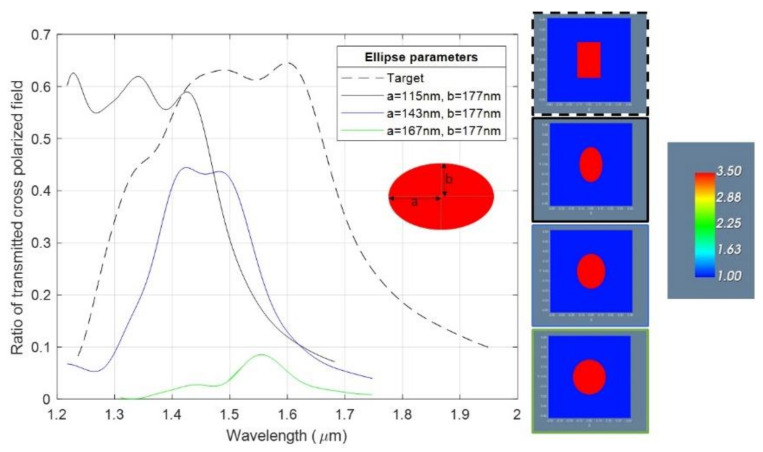
Plots of ellipse simulation curves, with different values for the a and b parameters based on metalenses patterns dimensions found from SEM images.

The measured UV-metalenses were those patterned with round corners, notated in Table 2 with “UV-metalens 3”. The selection was based on the cumulated previous experimental results and simulations, which concluded that a variation in length and width higher than 40 nm and a height around 800 nm could decrease the efficiency by up to 40% [4,47], due to the fact that the one near the w and l target values were presenting imprints defects due to the master-trapped pillar in the stamp. In order to explain the low efficiency for the NIL metalenses, we simulated it using the finite difference time domain (FDTD) method the case, in which instead of a rectangular shape (Figure 11—dashed line) the pillars had an oval shape. The first simulation parameters for the ellipse were for the target dimension (w = 2 × a = 230 nm; l = 2 × b = 354 nm), showing that even when the cross-section of the pillars is not perfectly rectangular, the efficiency should be near the theoretical one (Figure 11—black continuous line). The low efficiency of the UV-NIL metalens is given, according to the simulations (Figure 11—green line), by the nearly round shape of the pillars, which implies a decrease in the cross-polarization efficiency.

**Figure 12:**
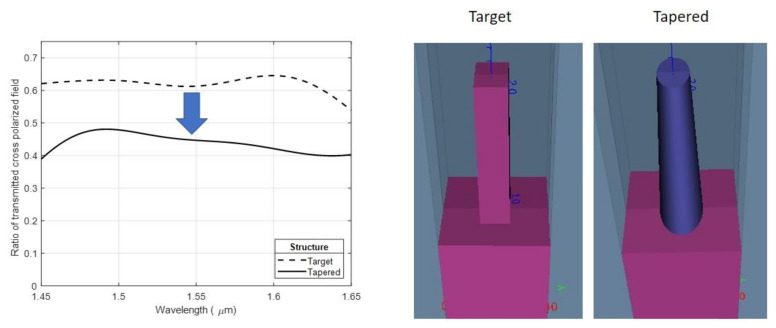
Plots of simulation curves considering the cases in which the patterns present straight (target) or tapered profile.

In the case of the EBL metalenses (see Figure 10c), the decrease in the efficiency (from the theoretical 60% to 45%) can be given by the pillars shape characterized by: (i) a small rectangular “hat” on top followed by a short neck and after going down with rounded corners similar with the simulation highlighted by the blue continuous line (Figure 11) and a (ii) slightly tapered profile. According to the simulations (see Figure 12), the tapered profile can induce a further decrease in the transmittivity and therefore a decrease in the lens efficiency. As discussed in Section 2.1, replacing the silicon substrate with a quartz substrate can raise the theoretical efficiency limit to 95% for perfectly vertical and rectangular pillars.

## 4. Conclusions

This study considered the entire process of fabricating metasurfaces by methods compatible with high-throughput production: from (i) master-fabrication by electron beam lithography (EBL); then (ii), pattern transfer using nanoimprint lithography (NIL); and thereafter (iii), deep reactive ion etch (DRIE) using cryogenic etching. The main challenges and critical parameters have been identified for achieving a successful process.

The silicon metasurface master fabrication was performed by EBL, followed by the cryogenic etching process of the silicone substrate. The optimization of the etching process was achieved by adjusting the O_2_ flow, in order to obtain vertical profiles, and by evaluation of different masking materials for reliable transfer of nanopatterns to the silicone substrate. The resultant structures present nearly vertical sidewalls with a small under-etch at the top of the pillars. This effect may be caused by a nonuniform heating gradient induced along the vertical axis of the pillars by the ICP power applied to the plasma. For the case of cryoetching, the NIL imprinted wafers with mr-NIL210 resist (500 nm), and the silicon etching depth was not influenced by the nonuniform heights of the mask resist pillars but rather by the incomplete removal of the residual layer. The highest lens efficiency of around 45% was achieved by using the EBL technique to pattern the etch mask. The discrepancy of around 16% from the simulated theoretical limit of 61% for silicon pillars on a silicon substrate may be largely attributed to two effects related to process imperfections: (i) the tapering of the fabricated structures serves to reduce the transmissivity, and (ii) the rounding of the structures reduces the efficiency at which the metasurface converts between circular polarization states. The total efficiency of the metalens is the product of the transmission and cross-polarization efficiency, and hence, both effects contribute to reducing the efficiency. Apart from these loss-mechanisms, there is also the presence of diffraction loss due to lateral variations among the metasurface elements not accounted for in the simulations. The low efficiency (6%) obtained in the case of NIL, despite having the same spot profile as the EBL metalenses, is related to various fabrication issues. In particular: (i) slight tapering in the sidewalls of the master structures implies a starting efficiency for the NIL metalenses of around 45%; (ii) the height of the pillars for the master template should have been lower that the height target dimensions of the metalenses in order to achieve a complete filling of the stamp cavities (the height should be 800 nm or lower for 1.2 μm tall target structures); and (iii) the residual layer was too thick, making its removal challenging by the RIE isotropic process. This, in turn, led to the rounding of the patterns. The relevant parameters for raising the lens efficiencies through process optimization have thus been identified.

## Figures and Tables

**Table 1:** Cryogenic process parameters.

ICP Power	RF Power	Pressure	SF_6_ Flow	O_2_ Flow	Table Temperature
1200 W	3 W	7.5 mTorr	60 sccm	8 sccm	−115 °C

**Table 2:** Dimensions of the metalenses patterns.

Sample	Dimensions			Deviation From Target		
	Height-h (µm)	Width-w (nm)	Length-l (nm)	Height (%)	Width (%)	Length (%)
Target	1200	230	354	-	-	-
EBL metalens	1200	236.8	355.2	0	−2.96	−0.33
Stamp 1	1083	224.2	340.4	9.75	2.52	3.84
UV-NIL metalens 1	842	235.9	312.6	29.83	−2.57	11.19
UV-NIL metalens 2	1066	210.3	302.6	11.16	8.56	14.52
UV-NIL metalens 3	1280	247.2	307	−6.66	−7.48	13.27

## Data Availability

The data of this study are available from the corresponding author upon reasonable request.
